# P-1097. Efficacy of Rifaquizinone in a Murine Model of Prosthetic Joint Infection (PJI) with Fluoroquinolone-Sensitive and -Resistant *Staphylococcus aureus*

**DOI:** 10.1093/ofid/ofae631.1285

**Published:** 2025-01-29

**Authors:** Mark E Pulse, Phung Nguyen, David Valtierra, Kelly Peterson, Zhenkun Ma, Huan Wang, William J Weiss

**Affiliations:** University of North Texas Health Science Center, Fort Worth, Texas; University of North Texas Health Science Center, Fort Worth, Texas; HSC College of Pharmacy, University of North Texas, Fort Worth, Texas; HSC College of Pharmacy, University of North Texas, Fort Worth, Texas; TenNor Therapeutics, Suzhou Industrial Park, Jiangsu, China (People's Republic); TenNor Therapeutics (Suzhou) Ltd, Suzhou, Jiangsu, China (People's Republic); University of North Texas Health Science Center, Fort Worth, Texas

## Abstract

**Background:**

Rifaquizinone (RFQ, TNP-2092) is a novel multitargeting drug conjugate in development for the treatment of serious or life-threatening bacterial infections including those caused by gram-positive pathogens that have developed or acquired resistance to commonly used antibiotics and those associated with medical devices. RFQ exerts its antibacterial activity by inhibiting RNA polymerase, DNA gyrase, and topoisomerase IV. Current studies sought to evaluate the therapeutic efficacy of RFQ following intraperitoneal (IP) administration for 7- to14-day treatment in a murine model of *S. aureus* prosthetic joint infection (PJI).

**Methods:**

K-wire was implanted in right hindlimb femurs of C57/BL6 mice followed by infection with 6.5-6.9 log_10_ CFU of a fluoroquinolone-sensitive (UNT005-4) or -resistant (UNT002-3) *S. aureus* strain. Doses of RFQ, ciprofloxacin, vancomycin, and vehicle were IP administered BID for 7 or 14 consecutive days starting 7 or 21 days after infection. Infected wires and femurs were collected and processed for CFU counts from untreated groups at the start of treatment and treated/vehicle groups within 18 hours of the final administered dose.

**Results:**

In the 7 day UNT005-4 infection study, mean wire and femur counts for the infection group were 4.77 log_10_ CFU and 7.89 log_10_ CFU on Day 7 and for vehicle treatment were 5.02 log_10_ CFU and 6.83 log_10_ CFU on Day 14. Comparatively, RFQ at doses of 10, 25, and 62.5 mg/kg for 7 days treatment significantly reduced mean wire and femur counts by > 2.40 and > 1.60 log_10_ CFU. In the 21 day UNT005-4 infection study, compared to the infection and vehicle treatment, the same doses of RFQ administered for 14 days significantly reduced mean counts by >1.02 log_10_ CFU for wires and > 0.88 log_10_ CFU for femurs. In the 21 day UNT002-3 infection study, RFQ administered for 14 days significantly reduced mean counts by > 1.50 log_10_ CFU for wire and > 0.9 log_10_ CFU for femur. As expected, reduction in mean counts following 7 or 14 days of ciprofloxacin and vancomycin treatments were less than RFQ.

**Conclusion:**

RFQ IP administered doses significantly reduced wire- and femur-associated counts for both *S. aureus* strains, and despite UNT002-3 resistant to ciprofloxacin treatment, the RFQ remained efficacious in the murine PJI model.

**Disclosures:**

**Mark E Pulse, MS**, TenNor Therapeutics (Suzhou) Ltd: Investigator **Phung Nguyen, BS**, TenNor Therapeutics (Suzhou) Ltd: Investigator **David Valtierra, Master**, TenNor Therapeutics (Suzhou) Ltd: Investigator **Kelly Peterson, PhD**, TenNor Therapeutics (Suzhou) Ltd: Investigator **Zhenkun Ma, PhD**, TenNor Therapeutics (Suzhou) Ltd: Employee **Huan Wang, PhD**, TenNor Therapeutics (Suzhou) Ltd: Employee **William J Weiss, MS**, TenNor Therapeutics (Suzhou) Ltd: Investigator

